# Neutrophil to high-density lipoprotein cholesterol ratio predicts left ventricular remodeling and MACE after PCI in patients with acute ST-segment elevation myocardial infarction

**DOI:** 10.3389/fcvm.2025.1497255

**Published:** 2025-04-03

**Authors:** Jianlin Chen, Anbang Liu, Dan Zhang, Tingting Meng, Xinhe Zhang, Weihong Xu, Yan Zheng, Guohai Su

**Affiliations:** ^1^ School of Clinical Medicine, Shandong Second Medical University, Weifang, China; ^2^ Shandong First Medical University & Shandong Academy of Medical Sciences, Jinan, Shandong, China; ^3^ Department of Cardiovascular Medicine, Jinan Central Hospital, Jinan, Shandong, China; ^4^ Research Center of Translational Medicine, Central Hospital Affiliated to Shandong First Medical University, Jinan, Shandong, China

**Keywords:** myocardial infarction, ventricular remodeling, neutrophil to high-density lipoprotein cholesterol ratio, biomarker, major adverse cardiovascular events

## Abstract

**Background:**

The neutrophil to high-density lipoprotein cholesterol ratio (NHR) has been proposed as a potential marker for predicting cardiovascular events. However, its prognostic role following percutaneous coronary intervention (PCI) in patients with acute ST-segment elevation myocardial infarction (STEMI) remains unclear. This study aimed to evaluate the predictive value of NHR for left ventricular remodeling (LVR) and long-term outcomes in STEMI patients post-PCI.

**Methods:**

This retrospective study included 299 STEMI patients who underwent PCI and were followed for 24 months post-procedure. Echocardiography was performed upon admission and at 6 months post-myocardial infarction (MI). LVR was defined as an increase in left ventricular diastolic volume (LVEDV) of at least 20% from baseline. Based on their VR status, patients were divided into LVR (*n* = 81) and non-LVR (*n* = 218) groups and clinical data were compared. A weighted logistic regression model was used to study the correlation between NHR and LVR. Weighted Cox proportional risk models were used to estimate hazard ratios (HRs) and 95% confidence intervals (95% CIs) for major adverse cardiovascular events (MACE). And the NHR was analyzed using receiver operating characteristic (ROC) curves to predict the occurrence of postoperative LVR and MACE in STEMI patients. Restricted cubic spline (RCS) analysis was used to explore the linear or non-linear relationship between NHR and LVR or MACE. Cox survival analysis was used to assess the relationship between NHR, LVR and survival time.

**Results:**

Among the 299 STEMI patients enrolled in the study, LVR was observed in 81 patients after 24 months of follow-up. The LVR group had significantly higher NHR levels compared to the non-LVR group (8.19 ± 1.95 vs. 6.23 ± 1.91, *P* < 0.001). After adjusting for potential confounders, a significant positive correlation was found between NHR and LVR. Each standard deviation increase in NHR was associated with a 43% higher risk of MACE (HR: 1.43, 95% CI: 1.25–1.64, *P* < 0.001). ROC curve analysis demonstrated that NHR could predict both LVR (AUC: 0.762) and MACE (AUC: 0.722). An NHR cut-off value of >8.13 was significantly linked to an increased risk of MACE (HR: 4.30, 95% CI: 2.41–7.69).

**Conclusions:**

NHR is an independent predictor of LVR and MACE after PCI in STEMI patients. Monitoring NHR may aid in identifying high-risk patients early, facilitating individualized treatment.

## Introduction

Acute ST-segment elevation myocardial infarction (STEMI) represents a severe manifestation of coronary artery disease, characterized by high mortality and morbidity rates. Despite significant improvements in the management of acute coronary syndromes (ACS), left ventricular remodeling (LVR) continues to be a critical determinant of long-term outcomes in STEMI patients (1). LVR refers to a series of unfavorable changes in the structure and function of the left ventricle following myocardial infarction, including ventricular dilatation, myocardial hypertrophy, and fibrosis, which can lead to heart failure and an increase the risk of recurrent cardiovascular events (2).

After MI, the inflammatory response is thought to play a crucial role in left ventricular remodeling. The infiltration and activation of neutrophil, the main effector cells of the acute inflammatory response, in the infarcted area leads to further myocardial injury and fibrosis (3). HDL-C not only has anti-atherosclerotic effects but also protects cardiovascular tissues, such as vascular endothelial cells and smooth muscle cells, through mechanisms including anti-inflammatory and antioxidant actions, as well as the promotion of reverse cholesterol transport (4, 5). Previous studies have shown that both increased neutrophil and decreased HDL-C are risk factors for cardiovascular disease (6), and the NHR is a new biomarker of lipid metabolism and inflammation (7). Therefore, the role of NHR in ventricular remodeling after myocardial infarction may reflect the balance between the body's inflammatory state and lipid metabolism, and has the advantages of rapid access, low cost and higher accuracy than other inflammatory biomarkers (8, 9). Numerous studies in recent years have confirmed the value of NHR in the prediction of cardiovascular disease occurrence, progression and prognosis (10–12). Nevertheless, the role of NHR in left ventricular remodeling after acute STEMI has not been fully investigated and validated. Therefore, our aim was to investigate the role of NHR in predicting LVR correlation in patients with acute STEMI treated with PCI in the prognostic studies.

## Methods

### Participants

**Figure 1:**
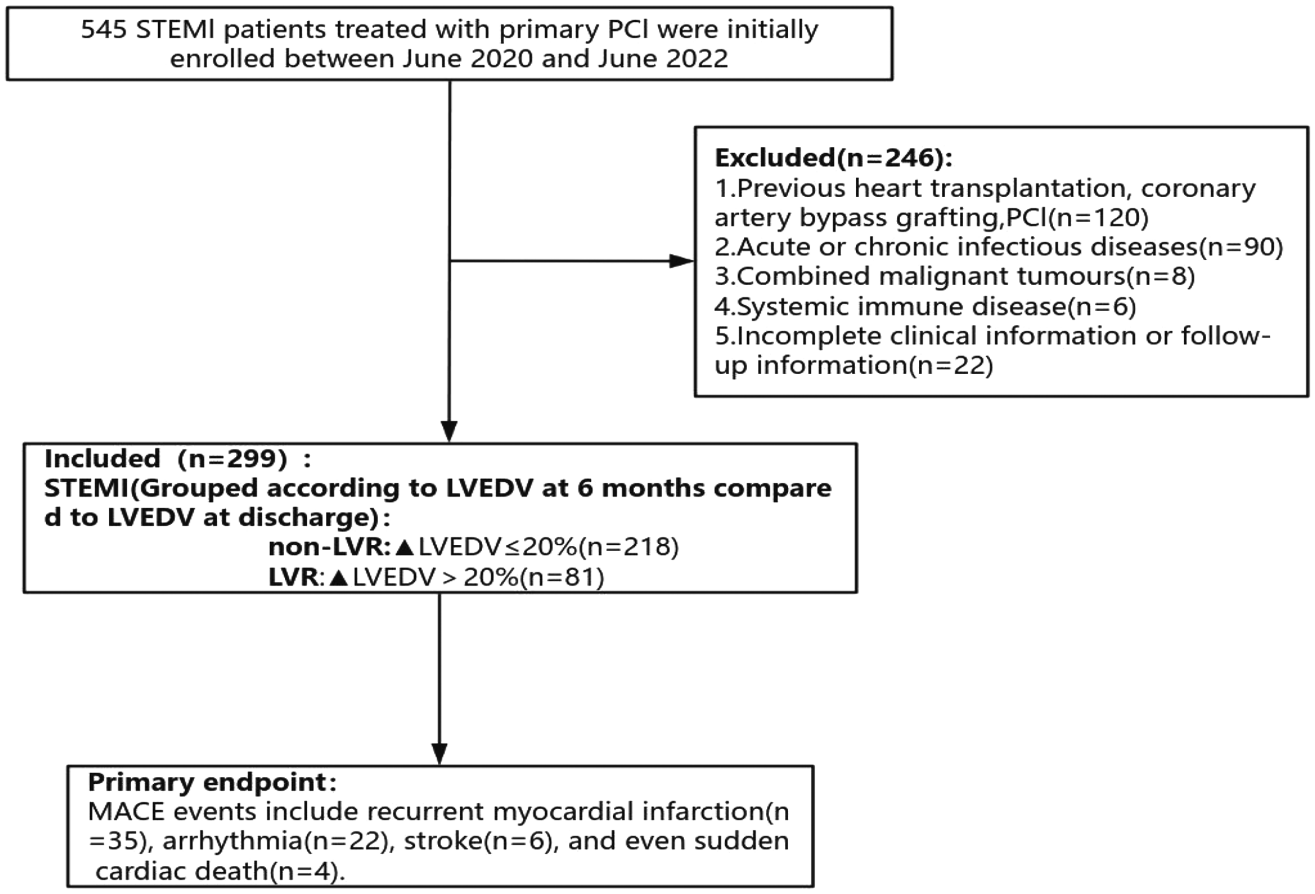
Flow chart for inclusion and exclusion of the study population.

Two hundred and ninety-nine patients with acute STEMI with PCI admitted to Jinan Central Hospital from June 2020 to June 2022 were selected, all of whom met the diagnostic criteria for STEMI in the 2023 ESC Guidelines (13) and were diagnosed with coronary angiography (CAG), and all of whom underwent PCI within 12 h of the onset of the disease. The exclusion criteria were: (1) Previous heart transplantation, coronary artery bypass grafting, PCI; (2) Acute or chronic infectious diseases; (3) Combined malignant tumors; (4) Systemic immune disease; (5) Incomplete clinical information or follow-up information. All patients were divided into 81 cases in the LVR group and 218 cases in the non-LVR group according to the occurrence of LVR. The study was reviewed and approved by the Medical Ethics Committee of Jinan Central Hospital (Ethical Review Number: 20240901001). All patients and their families were informed about the study and provided written informed consent. Patient enrollment and study design were shown in Figure 1.

### Diagnostic criteria

Diagnostic criteria for LVR an increase of >20% in LVEDV over LVEDV at discharge within 6 months of follow-up is ed as LVR (14).

### Clinical follow-up and study end points

In this 24-month follow-up study of 299 acute STEMI patients post-PCI, we assessed LVR, defined as a >20% increase in LVEDV index, and major adverse cardiovascular events (MACE), MACE was defined as a composite of recurrent myocardial infarction, arrhythmia, stroke, and sudden cardiac death during the follow-up period (15). Endpoints were evaluated using standardized criteria by a blinded clinical events committee, ensuring rigorous data collection and analysis.

### Data collection

The current study evaluated demographic data from medical records, including age, gender, smoking status, and medical history (e.g., history of hypertension and diabetes). Heart rate on admission, procedural status (culprit vessel, infarct site, initial blood flow, terminal blood flow, total stent length, and procedure time) were collected, and information on medication during hospitalization was also included. Initial blood flow and terminal blood flow refer to pre- and post-interventional Thrombolysis in Myocardial Infarction (TIMI) flow grades, as evaluated during coronary angiography (16). All patients underwent one routine blood test as per immediately after admission. Laboratory parameters included complete blood count [white blood cell count (WBC), neutrophil count, lymphocyte count, monocyte count, platelet count (PLT)], lipids (triglycerides, total cholesterol, LDL-C, HDL-C) and markers of myocardial injury (cTnT), MACE and MACE time. In addition, we collected echocardiographic data (LVEF1, LVEDV1, LVESV1) were recorded during MI and six months post-MI (LVEF2, LVEDV2, LVESV2), as well as data related to the CAG (culprit vessel, infarct location, initial blood flow, terminal blood flow, operative time, total length).

### Statistical analysis

All statistical analyses were performed using SPSS software (version 25.0; SPSS Inc., Chicago, IL, USA) and R Studio software (version 4.3.0). Continuous variables were presented as mean ± standard deviation (SD) or median with interquartile range (IQR) based on their distribution and were compared using an independent samples *t*-test or the Mann–Whitney *U*-test, as appropriate. Categorical variables were expressed as frequencies and percentages and were compared between groups using the chi-square test or Fisher's exact test when necessary. All statistical tests were two-sided, and a *P*-value of <0.05 was considered statistically significant.

To assess the relationship between the NHR and LVR, as well as MACE after PCI in patients with acute STEMI, a weighted logistic regression model was employed. This model was used to estimate odds ratios (ORs) and 95% confidence intervals (CIs) for the association between NHR and LVR. The Cox proportional hazards models were applied to calculate hazard ratios (HRs) and 95% CIs for the incidence of MACE, adjusting for potential confounders, including age, sex, smoking status, and comorbidities.

ROC curve analysis was conducted to evaluate the predictive ability of NHR for both LVR and MACE, with the area under the curve (AUC) values calculated to determine the discrimination ability of NHR as a predictor. The optimal cut-off value for NHR in predicting MACE was determined using the Youden index. Net Reclassification Improvement (NRI) and Integrated Discrimination Improvement (IDI) metrics were used to compare the predictive performance of NHR against neutrophil count alone. Logistic regression models were applied to assess associations between predictors and outcomes, with Akaike Information Criterion (AIC) and Bayesian Information Criterion (BIC) used to evaluate model fit.

RCS analysis was performed to explore potential non-linear relationships between NHR and both LVR and MACE, providing a flexible method for visualizing the dose-response relationship. Kaplan–Meier survival analysis was used to compare survival curves for different NHR groups, and the log-rank test was applied to assess differences in survival distributions.

## Results

### Characteristics of the study participants

A comparison of the baseline characteristics is shown in Table 1. Patients with LVR had higher NHR, Platelets, Neutrophil, Lymphocyte, TG, cTnT, and WBC levels compared with the non-LVR group (*P* < 0.05, for all). Additionally, patients in the LVR group had lower LVEF2, higher LVEDV2, LVESV2, and a higher incidence of MACE compared with the non-LVR group (*P* < 0.05, for all).

**Table 1:** Baseline characteristics of participants.

Group	Total (*n* = 299)	Non-LVR (*n* = 218)	LVR (*n* = 81)	*P*-value
Age (years)	62.05 ± 12.94	61.71 ± 12.54	62.98 ± 13.99	0.452
HR (/min)	77.17 ± 17.41	76.28 ± 17.27	79.57 ± 17.67	0.147
NHR	6.76 ± 2.11	6.23 ± 1.91	8.19 ± 1.95	<0.001
cTnT (ng/ml)	939.70 ± 1,590.87	788.89 ± 1,336.80	1,345.59 ± 2,085.99	0.007
Platelets (×109/L)	236.99 ± 118.44	236.42 ± 134.04	238.54 ± 59.31	0.891
WBC (×1,012/L)	9.42 ± 2.46	9.02 ± 2.21	10.48 ± 2.79	<0.001
Neutrophil (×109/L)	7.14 ± 2.04	6.66 ± 1.84	8.45 ± 1.97	<0.001
Lymphocyte (×109/L)	2.94 ± 1.47	2.70 ± 1.45	3.58 ± 1.31	<0.001
Monocyte (×109/L)	0.77 ± 0.63	0.86 ± 0.70	0.55 ± 0.24	<0.001
HDL (mmol/L)	1.09 ± 0.25	1.10 ± 0.23	1.07 ± 0.29	0.366
LDL (mmol/L)	2.68 ± 0.78	2.64 ± 0.83	2.80 ± 0.61	0.109
TC (mmol/L)	4.24 ± 1.09	4.27 ± 1.08	4.16 ± 1.13	0.466
TG (mmol/L)	2.14 ± 0.68	1.95 ± 0.54	2.65 ± 0.77	<0.001
LVESV1 (ml)	47.22 ± 14.43	48.42 ± 13.95	43.98 ± 15.26	0.018
LVEDV1 (ml)	94.32 ± 24.13	94.17 ± 24.56	94.73 ± 23.05	0.858
LVEF1 (%)	56.60 ± 6.76	56.20 ± 5.98	57.67 ± 8.47	0.095
LVESV2 (ml)	55.96 ± 17.69	51.61 ± 14.76	67.68 ± 19.58	<0.001
LVEDV2 (ml)	114.51 ± 34.70	101.44 ± 25.09	149.68 ± 32.56	<0.001
LVEF2 (%)	53.87 ± 9.06	57.64 ± 6.38	43.73 ± 7.25	<0.001
Operative Time (min)	52.23 ± 20.33	52.03 ± 20.55	52.78 ± 19.84	0.777
Total length (mm)	26.00 ± 16.57	26.28 ± 16.87	25.23 ± 15.83	0.627
MACE Time (month)	9.75 ± 6.23	10.45 ± 5.77	7.88 ± 7.05	0.001
Sex				
Female	69 (23.08%)	48 (22.02%)	21 (25.93%)	0.476
Male	230 (76.92%)	170 (77.98%)	60 (74.07%)	
Infarction location				
Anterior wall	137 (45.82%)	100 (45.87%)	37 (45.68%)	0.942
Inferior wall	153 (51.17%)	111 (50.92%)	42 (51.85%)	
Lateral wall	9 (3.01%)	7 (3.21%)	2 (2.47%)	
Culprit vessel				
RCA	136 (45.48%)	99 (45.41%)	37 (45.68%)	0.876
LAD	153 (51.17%)	111 (50.92%)	42 (51.85%)	
LCX	10 (3.34%)	8 (3.67%)	2 (2.47%)	
Smoking				
No	127 (42.47%)	93 (42.66%)	34 (41.98%)	0.915
Yes	172 (57.53%)	125 (57.34%)	47 (58.02%)	
Drinking				
No	163 (54.52%)	115 (52.75%)	48 (59.26%)	0.315
Yes	136 (45.48%)	103 (47.25%)	33 (40.74%)	
Hypertension				
No	124 (41.47%)	95 (43.58%)	29 (35.80%)	0.225
Yes	175 (58.53%)	123 (56.42%)	52 (64.20%)	
Diabetes				
No	220 (73.58%)	162 (74.31%)	58 (71.60%)	0.637
Yes	79 (26.42%)	56 (25.69%)	23 (28.40%)	
Initial blood flow				
0	177 (59.20%)	118 (54.13%)	59 (72.84%)	0.022
1	43 (14.38%)	33 (15.14%)	10 (12.35%)	
2	34 (11.37%)	28 (12.84%)	6 (7.41%)	
3	45 (15.05%)	39 (17.89%)	6 (7.41%)	
Terminal blood flow				
0	1 (0.33%)	0 (0.00%)	1 (1.23%)	0.007
2	7 (2.34%)	2 (0.92%)	5 (6.17%)	
3	291 (97.32%)	216 (99.08%)	75 (92.59%)	
Aspirin				
No	15 (5.02%)	9 (4.13%)	6 (7.41%)	0.248
Yes	284 (94.98%)	209 (95.87%)	75 (92.59%)	
Statin				
No	12 (4.01%)	8 (3.67%)	4 (4.94%)	0.619
Yes	287 (95.99%)	210 (96.33%)	77 (95.06%)	
Clopidogrel				
No	183 (61.20%)	141 (64.68%)	42 (51.85%)	0.043
Yes	116 (38.80%)	77 (35.32%)	39 (48.15%)	
Ticagrelor				
No	85 (28.43%)	55 (25.23%)	30 (37.04%)	0.044
Yes	214 (71.57%)	163 (74.77%)	51 (62.96%)	
MACE				
No	232 (77.59%)	206 (94.50%)	26 (32.10%)	<0.001
Yes	67 (22.41%)	12 (5.50%)	55 (67.90%)	

All values are presented as mean ± SD or as counts (weighted proportion). *P*-value: obtained by Kruskal Wallis rank sum test for continuous variables, and Fisher's exact probability test for count variables with theoretical number <10.

### Associations between NHR and the occurrence of LVR or MACE

**Figure 2:**
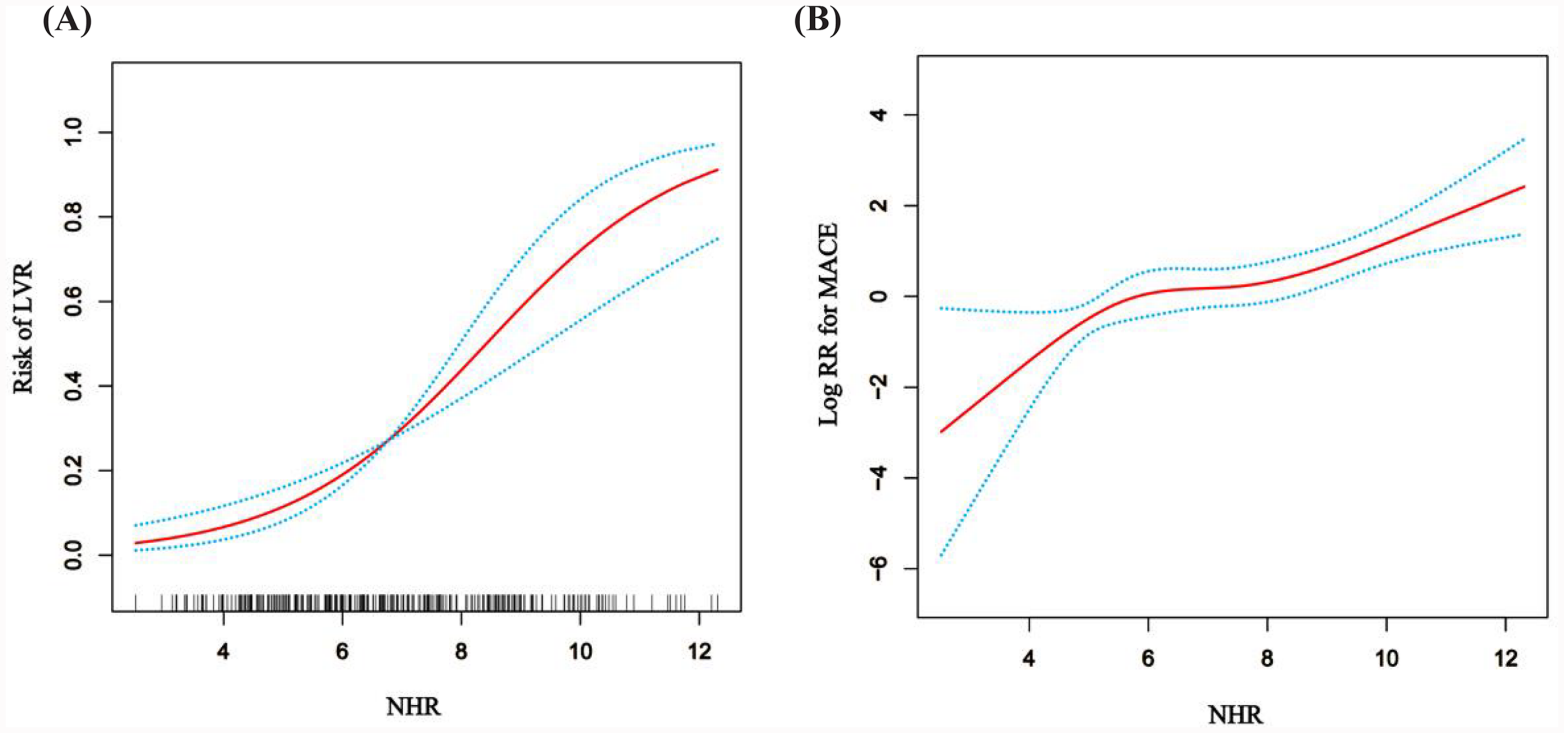
The general additive model (GAM) with restricted cubic splines (RCS) illustrates the relationship between NHR and LVR or MACE. **(A)** Association between NHR and log RR for LVR. **(B)** Association between NHR and logRR for MACE. The model was adjusted for Age, Smoking, Sex, Drinking, Infarction location, culprit vessel, Hypertension, Diabetes, cTnT, Aspirin, Statin, Clopidogrel, Ticagrelor, LVEF1, LVESV1, LVEDV1, LVEF2, LVESV2 and LVEDV2.

**Figure 3:**
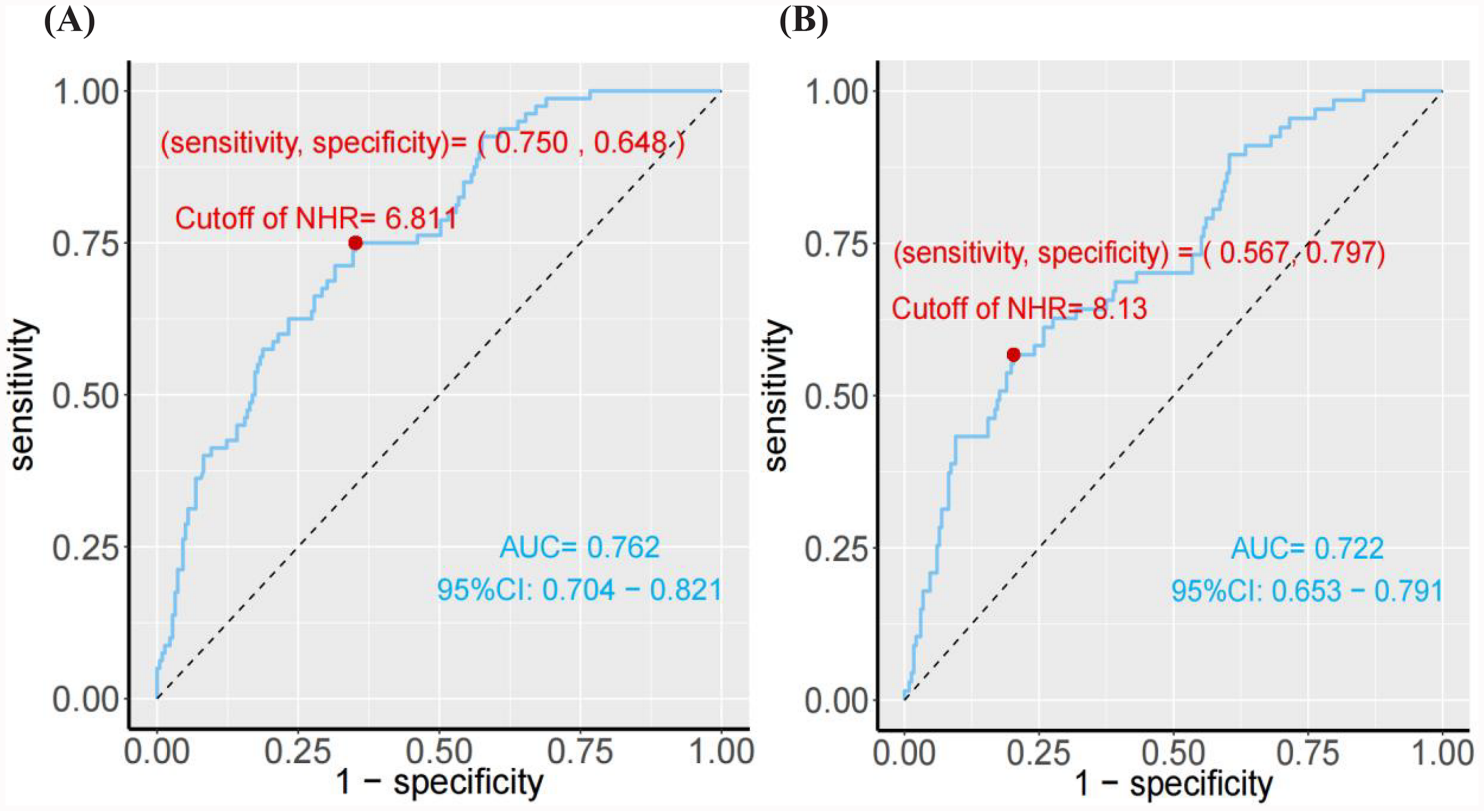
**(A)** The ROC curve analysis of NHR for predicting the presence of ventricular remodeling. **(B)** The ROC curve analysis of NHR for predicting the MACE.

The study revealed a strong association between the higher NHR and the development of LVR in patients who underwent PCI for acute STEMI. As shown in Table 1, the LVR group (*n* = 81) had significantly higher NHR levels (8.19 ± 1.95) compared to the non-LVR group (6.23 ± 1.91), with a *P*-value<0.001, indicating a substantial difference. Furthermore, multivariate regression analysis, controlling for confounders like age, smoking, infarction location, and medication, demonstrated that for every standard deviation increase in NHR, the OR for LVR was 1.82 (95% CI: 1.45–2.27), confirming NHR as an independent predictor of LVR (Table 2). To further explore the effect of HDL-C alone on LVR and MACE in NHR, we performed stratified regression analyses (Supplementary Table S1). The results showed that HDL-C as a variable alone had limited predictive value for LVR and MACE. After adjusting for confounders, the association of HDL-C on LVR did not reach statistical significance (adjusted OR: 0.56; 95% CI: 0.09–3.36; *P* = 0.52), and the association on MACE was similarly insignificant (adjusted HR: 0.47; 95% CI: 0.15–1.48; *P* = 0.20). To visually assess the relationship between NHR and LVR, Figure 2A, employs an RCS model, highlighting a non-linear association. This figure illustrates that the risk of LVR increases exponentially as NHR levels rise beyond a certain threshold, reinforcing the statistical findings. Additionally, the ROC curve analysis in Figure 3A, further supports the predictive capability of NHR for LVR, with an AUC of 0.762, indicating moderate discrimination. These combined statistical and graphical insights emphasize the clinical importance of monitoring NHR in predicting LVR post-PCI.

**Table 2:** Associations between NHR and LVR.

Exposure	Model 1	Model 2	Model 3
NHR	1.63 (1.41, 1.89) ***	1.67 (1.44, 1.95) ***	1.82 (1.45, 2.27) ***
NHR (per SD increase)	2.80 (2.06, 3.81) ***	2.96 (2.14, 4.09) ***	3.53 (2.20, 5.65) ***

All values are presented as OR (95% CI); *P*-value: ***P < 0.001.

Model 1: Unadjusted.

Model 2: Adjusted for Age, Smoking, Sex and Drinking.

Model 3: Adjusted for Age, Smoking, Sex, Drinking, Infarction location, culprit Vessel, Hypertension, Diabetes, cTnT, Aspirin, Statin, Clopidogrel, Ticagrelor, LVEF1, LVESV1, LVEDV1, LVEF2, LVESV2, and LVEDV2.

**Figure 4:**
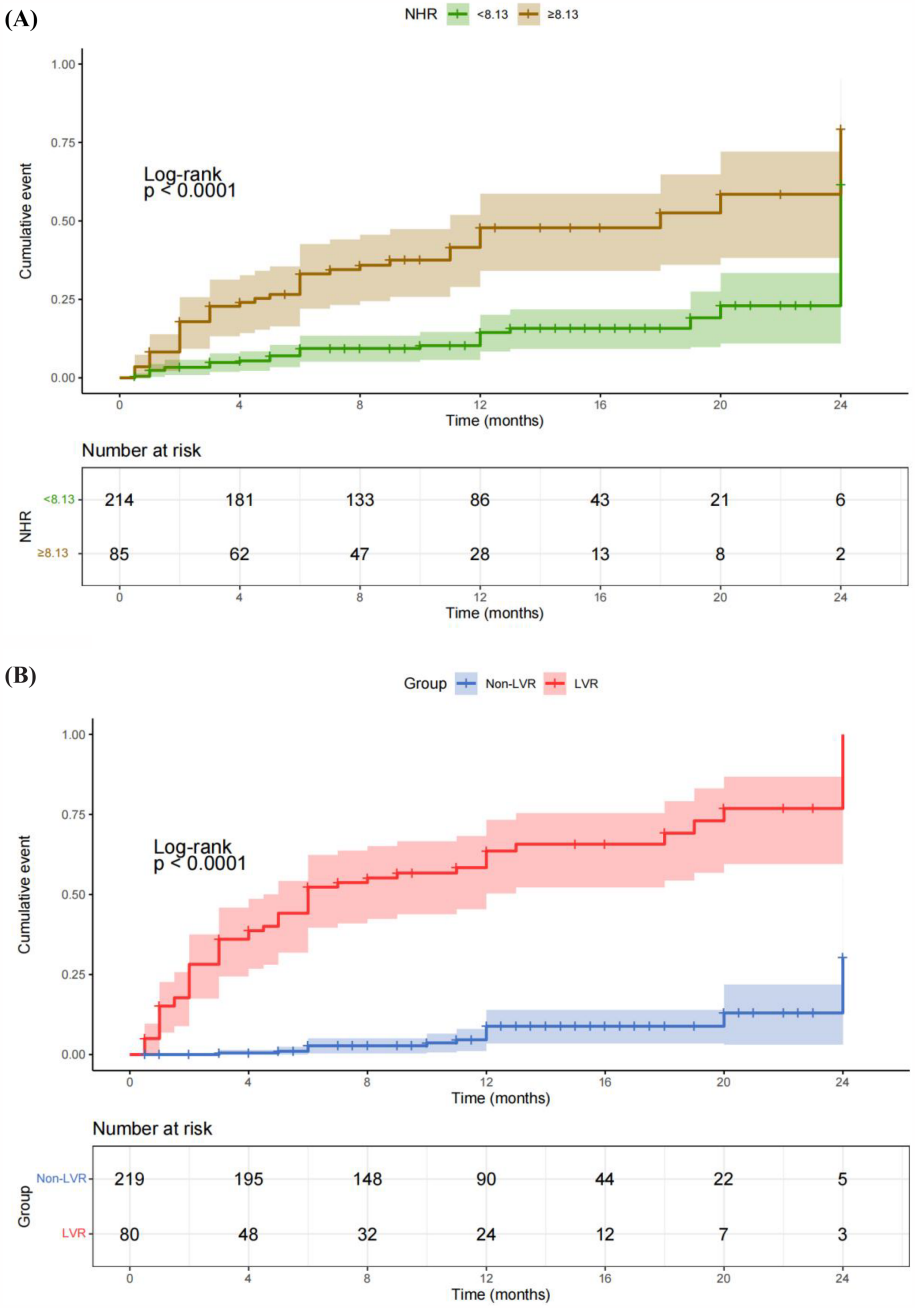
Kaplan–Meier survival curves illustrating the relationships of NHR and LVR with the cumulative incidence of MACE. **(A)** Kaplan–Meier survival curves showing the correlation between NHR and the cumulative incidence of MACE. Patients with elevated NHR levels had a significantly higher cumulative incidence of MACE compared to those with lower NHR levels. **(B)** Kaplan–Meier survival curves demonstrating the correlation between LVR and cumulative MACE. Patients who developed LVR exhibited a significantly higher cumulative incidence of MACE compared to those without LVR.

The study also demonstrated a significant association between elevated NHR and the occurrence of MACE in patients post-PCI. As shown in Table 3, the HR for MACE was 1.43 (95% CI: 1.25–1.64) for each standard deviation increase in NHR, adjusted for factors such as age, smoking status, comorbidities, and medication. Moreover, patients with NHR levels ≥8.13 were at a significantly higher risk of MACE, with an adjusted HR of 4.30 (95% CI: 2.41–7.69), compared to those with lower NHR levels. This underscores the strong predictive value of NHR in determining long-term adverse cardiovascular outcomes. The predictive capability of NHR for MACE is further confirmed by the ROC curve analysis in Figure 3B, which shows an AUC of 0.762. This indicates that NHR is a reliable marker for predicting the risk of MACE, with moderate discrimination ability. The non-linear dose-response relationship between NHR and MACE is also demonstrated in Figure 2B using an RCS model. The curve illustrates a clear increase in the HR for MACE as NHR rises, reinforcing the idea that higher NHR levels are associated with a greater likelihood of adverse cardiovascular events. Additionally, Figure 4B depicts Kaplan–Meier survival curves, highlighting that patients with elevated NHR levels experience a higher cumulative incidence of MACE over time compared to those with lower NHR. This combination of statistical data and visual evidence emphasizes the clinical importance of NHR as a biomarker for predicting MACE in STEMI patients post-PCI and suggests that close monitoring of NHR could guide risk stratification and intervention strategies.

**Table 3:** HRs (95% CIs) for MACE according to the NHR.

Exposure	Model 1	Model 2	Model 3
NHR	1.39 (1.24, 1.56) ***	1.38 (1.23, 1.55) ***	1.43 (1.25, 1.64) ***
NHR (per SD increase)	2.01 (1.58, 2.56) ***	1.98 (1.56, 2.51) ***	2.12 (1.60, 2.82) ***
NHR <8.13	1 (Reference)	1 (Reference)	1 (Reference)
NHR ≥8.13	3.68 (2.27, 5.97) ***	3.95 (2.40, 6.48) ***	4.30 (2.41, 7.69) ***

All values are presented as HR (95% CI); *P*-value: ***P < 0.001.

Model 1: Unadjusted.

Model 2: Adjusted for Age, Smoking, Sex and Drinking.

Model 3: Adjusted for Age, Smoking, Sex, Drinking, Infarction location, culprit Vessel, Hypertension, Diabetes, cTnT, Aspirin, Statin, Clopidogrel, Ticagrelor, LVEF1, LVESV1, LVEDV1, LVEF2, LVESV2, and LVEDV2.

### Contribution of neutrophils and HDL-C in NHR for predicting LVR and MACE

To explore the individual contribution of neutrophils and HDL-C in NHR, we conducted additional analyses using the ROC curve, Net Reclassification Improvement (NRI), and Integrated Discrimination Improvement (IDI) analysis to compare the predictive capabilities of NHR and neutrophil count. The analysis revealed that neutrophils had higher predictive value for LVR and MACE, with AUCs of 0.747 and 0.691, respectively (Supplementary Figures S1C,D). In contrast, HDL-C demonstrated lower predictive value for these outcomes, with AUCs of 0.553 and 0.583, respectively (Supplementary Figures S1A,B). As shown in Supplementary Table S4, NRI and IDI analyses demonstrated that NHR significantly improved prediction for LVR (NRI = 0.1817, IDI = 0.0216) and MACE (NRI = 0.3883, IDI = 0.0424) compared to neutrophil count alone. Furthermore, as shown in Supplementary Table S6, logistic regression models incorporating NHR showed better performance with lower AIC and BIC values, confirming the superior predictive ability of NHR (*P* < 0.01, Likelihood Ratio Test). These findings indicate that the value of NHR in predicting LVR and MACE is superior to neutrophil count.

In addition, stratified analyses were performed to evaluate the impact of high neutrophil counts (≥8 × 10^9^/L) and low HDL-C levels (<1 mmol/L) on LVR and MACE outcomes. The results demonstrated a progressive increase in risk across subgroups, with the highest risk observed in patients with both high neutrophil counts and low HDL-C levels (Supplementary Tables S2, S3). This underscores the critical interplay between inflammation and lipid metabolism in determining cardiovascular outcomes.

We found that TIMI (pre-PCI to pos-PCI) values of 0–0 and 0–2 had higher median NHRs, while TIMI values of 1–3 and 2–3 had lower NHRs. Therefore, it is speculated that the smaller the improvement of TIMI (pre-PCI to pos-PCI), the higher the NHR value may be (Supplementary Tables S4). To some extent, NHR may have important predictive value for cardiac function and prognosis.

## Discussion

Acute STEMI remains a life-threatening condition. Although PCI is effective in restoring blood flow, the irreversible damage to myocardial cells is often underestimated (17). Given that cardiomyocytes cannot regenerate, patients are at an increased risk of developing LVR, a condition that may progress to significantly worsen outcomes (18). While various biomarkers have been identified to predict adverse prognosis following STEMI, the predictive accuracy of these markers has limitations (19).

Inflammation and lipid metabolism are central to the pathophysiology of LVR (20). Following MI, the inflammatory response, particularly mediated by neutrophil, plays a crucial role in ventricular remodeling. Neutrophil, as primary effector cells of acute inflammation, infiltrate the infarcted myocardium and release proteolytic enzymes and reactive oxygen species, thereby exacerbating myocardial damage and promoting fibrosis (21). HDL-C, in contrast, is known for its cardioprotective properties, including anti-inflammatory, antioxidative, and cholesterol transport functions (5, 22). HDL-C mitigates oxidative stress and reduces the inflammatory response, thus playing a critical role in protecting against adverse cardiovascular outcomes. Therefore, NHR, as a ratio that reflects both inflammatory activity and lipid metabolic status, serves as a useful integrated marker for cardiovascular risk.

In this study, NHR was significantly higher in the LVR group compared to the non-LVR group (8.19 ± 1.95 vs. 6.23 ± 1.91, *P* < 0.001) (Table 1). This finding is consistent with previous research indicating that higher NHR correlates with worse outcomes in cardiovascular diseases (23). Furthermore, ROC curve analysis (Figure 3A) demonstrated that NHR had moderate discriminatory power for predicting LVR, with an AUC of 0.762, supporting the potential of NHR as an early indicator of ventricular remodeling risk. Compared with previous studies (24, 25), the NHR had more predictive value than other studies establishing it of LVR biomarkers, including CRP (AUC = 0.61), BNP (AUC = 0.61), TnT (AUC = 0.66) and TGFBR1 (AUC = 0.72). Multivariate regression analysis revealed that for every standard deviation increase in NHR, the OR for LVR increased by 1.82 (95% CI: 1.45–2.27), underscoring NHR as an independent risk factor (Table 2). A further study was conducted to investigate which of neutrophils and NHR alone had higher predictive value. The results showed that although NHR was slightly higher than neutrophils in predicting LVR and MACE when analysed under the ROC curve, NHR significantly improved the predictive accuracy and discriminatory ability, and its advantage was reflected by the NRI and IDI metrics (LVR: NRI = 0.1817, IDI = 0.0216; MACE: NRI = 0.3883, IDI = 0.0424) (Supplementary Tables S5). In addition, the NHR-based logistic regression model performed better on model fit metrics such as AIC and BIC, and statistical tests showed that its predictive performance was significantly better than that of neutrophils alone (*P* < 0.01) (Supplementary Tables S6). These results suggest that NHR is able to predict LVR and MACE more accurately than a single inflammation marker by combining the dual roles of inflammation and lipid metabolism, and has a higher value for clinical application.

The association between NHR and MACE was equally compelling. Cox regression analysis indicated that for each standard deviation increase in NHR, the risk of MACE rose by 43% (HR: 1.43, 95% CI: 1.25–1.64, *P* < 0.001). Patients with NHR values ≥8.13 were at significantly higher risk of MACE (HR: 4.30, 95% CI: 2.41–7.69, *P* < 0.001) (Table 3). These results align with previous studies showing that elevated neutrophil counts and reduced HDL-C levels are independently associated with adverse cardiovascular outcomes (26). The ROC analysis (Figure 3B) confirmed the robustness of NHR as a predictor of MACE, with an AUC of 0.722. Additionally, the RCS analysis (Figure 2B) revealed a non-linear relationship between NHR and MACE, with a pronounced increase in MACE risk as NHR exceeded approximately 8.13, suggesting that elevated NHR significantly increases the likelihood of adverse outcomes.

The potential mechanisms by which NHR influences LVR and MACE likely involve the interplay between inflammation and lipid metabolism. Elevated neutrophil counts may contribute to sustained inflammation and subsequent myocardial fibrosis, while low HDL-C levels diminish the protective effects of cholesterol efflux and antioxidative functions. This imbalance between heightened inflammation and impaired lipid metabolism could explain why higher NHR levels are associated with poorer cardiovascular outcomes (27). Building on this, the results of this study highlight the prognostic significance of both NHR and LVR in predicting long-term outcomes in STEMI patients post-PCI. Figure 4A shows that patients with elevated NHR levels had a significantly higher cumulative incidence of MACE, reinforcing the role of inflammation and lipid metabolism in adverse cardiovascular outcomes. According to guidelines and previous studies (28, 29), we considered neutrophil counts ≥8 × 10^9^/L as high neutrophils and HDL-C <1 mmol/L as low HDL-C. Based on our ROC best cutoff results for NHR prediction of MACE, we considered NHR≥8.13 to be high (Figures 3B, 4A). Figure 4B demonstrates that patients with LVR experienced a higher cumulative incidence of MACE compared to those without LVR, emphasizing the detrimental impact of ventricular remodeling. Together, these findings suggest that integrating biomarkers like NHR with structural indicators such as LVR can enhance post-PCI risk assessment and guide early interventions to reduce MACE risk.

The differences in coronary flow between the LVR and non-LVR groups highlight the critical role of coronary perfusion in preventing adverse ventricular remodeling. The higher frequency of initial residual coronary flow in the non-LVR group suggests that better baseline coronary perfusion may reduce the extent of myocardial ischemia. Conversely, impaired coronary flow after PCI, observed more frequently in the LVR group, likely exacerbates ischemia-reperfusion injury and contributes to adverse remodeling. These findings similarly highlight the importance of achieving optimal coronary flow restoration during PCI to improve myocardial recovery and reduce the risk of LVR (30, 31). This highlights the need for integrated strategies that combine advanced reperfusion techniques with reliable prognostic tools to optimize outcomes in STEMI patients undergoing PCI.

## Clinical implications

The clinical implications of our findings are substantial. NHR is a simple, cost-effective, and easily measurable biomarker that can be obtained from routine blood tests, making it a feasible tool for early risk stratification in STEMI patients undergoing PCI (7, 32). Monitoring NHR could enable clinicians to identify high-risk patients early, allowing for more aggressive interventions to prevent LVR and subsequent MACE. Additionally, based on the non-linear relationship demonstrated in the RCS analysis (Figures 2A,B), clinicians may consider a threshold-based approach to guide therapeutic decision-making in patients with elevated NHR, with the goal of reducing cardiovascular risk.

## Limitations

Despite the promising results, this study has certain limitations. The retrospective nature and single-center design may limit the generalizability of the findings. Additionally, the relatively small sample size may introduce bias. Finally, while we identified a strong association between NHR and adverse outcomes, the underlying molecular mechanisms were not investigated in this study. Future research should focus on larger, prospective studies to validate these findings and further explore the biological mechanisms by which NHR influences LVR and MACE.

## Conclusion

In summary, as a new biomarker reflecting the balance between inflammation and metabolism, NHR is involved in the occurrence and development of LVR after PCI in STEMI patients, and it is an independent influence factor of LVR after PCI in STEMI patients. The clinic can assess the high-risk group according to the optimal cut-off value of NHR for predicting the occurrence of LVR in patients with PCI, and then give targeted preventive and curative measures.

## Data Availability

The original contributions presented in the study are included in the article/Supplementary Material, further inquiries can be directed to the corresponding authors.
