# Supplementary material for: Neutrophil to high-density lipoprotein cholesterol ratio predicts left ventricular remodeling and MACE after PCI in patients with acute ST-segment elevation myocardial infarction
Source: Front Cardiovasc Med. 2025 Apr 3;12:1497255. doi: 10.3389/fcvm.2025.1497255 (PMC12003286; doi:10.3389/fcvm.2025.1497255)
Supplement: Supplementary file 2 [file Datasheet2.pdf]

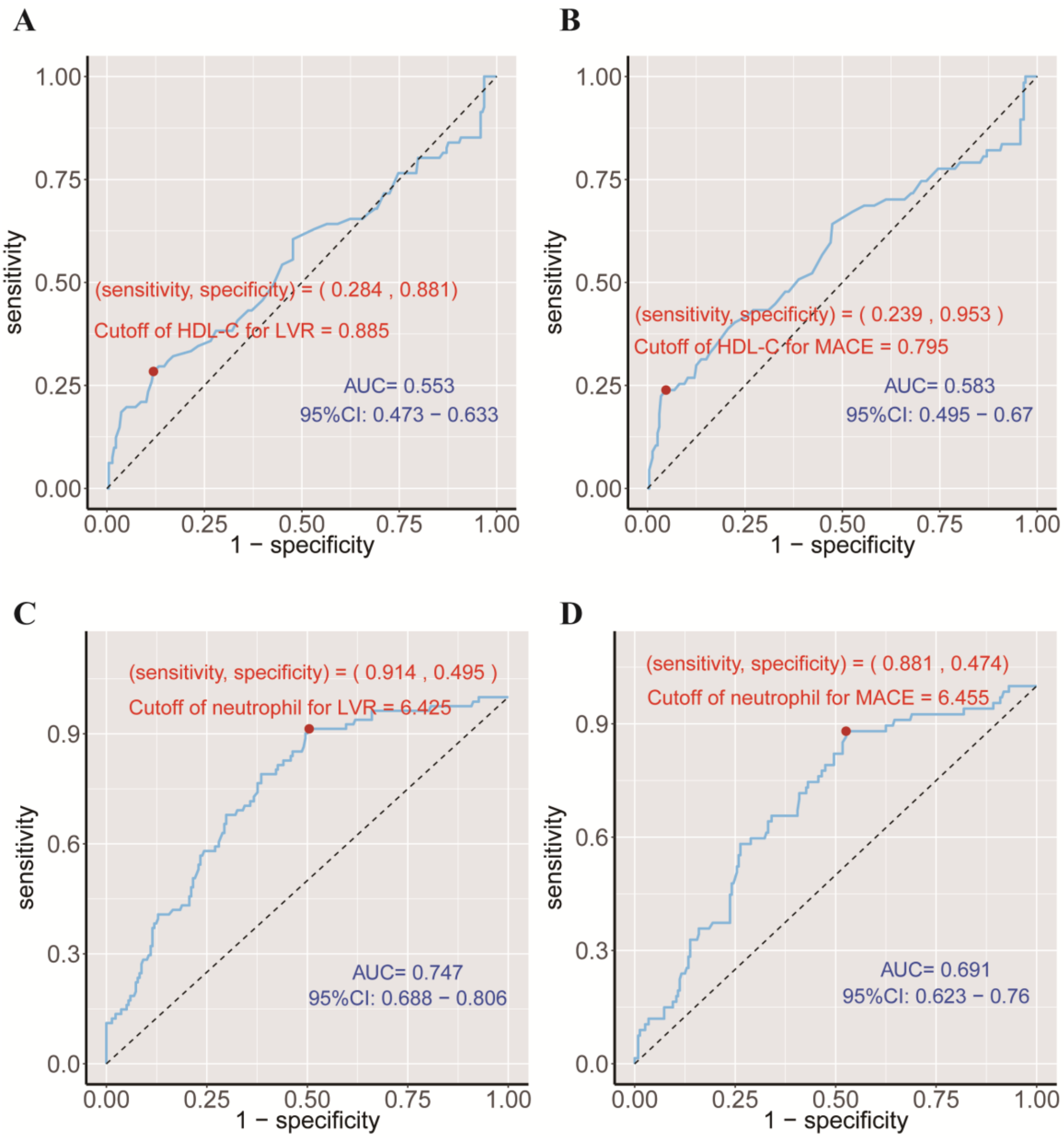

Figure S1. (A)The ROC curve analysis of HDL-C for predicting the presence of LVR. (B)The ROC curve analysis of HDL-C for predicting the MACE. (C)The ROC curve analysis of Neutrophil for predicting the presence of LVR. (D)The ROC curve analysis of Neutrophil for predicting the MACE.

Table S1. Associations of neutrophil and HDL-C with LVR and MACE

Exposure	Outcome	OR (95% CI) P-value		
		model 1	model 2	model 3
	LVR			
Neutrophil		1.62(1.40, 1.90) <0.001	1.62(1.40, 1.90) <0.001	1.71(1.45, 2.06) <0.001
HDL-C		0.61(0.20, 1.74) 0.37	0.56(0.18, 1.64) 0.3	0.56 (0.09, 3.36) 0.52
		HR (95% CI) P-value		
		model 1	model 2	model 3
	MACE			
Neutrophil		1.28(1.15, 1.43) <0.001	1.28(1.14, 1.42) <0.001	1.35(1.2, 1.52) <0.001
HDL-C		0.39(0.13, 1.17) 0.09	0.42(0.14, 1.27) 0.12	0.47(0.15, 1.48) 0.2

Model1: Unadjusted

Model2: Adjusted for Age, Smoking, Sex, and Drinking

Model3: Adjusted for Age, Smoking, Sex, Drinking, Infarction location, culprit Vessel, Hypertension, Diabetes, cTnT, Aspirin, Statin, Clopidogrel, Ticagrelor, LVEF1, LVESV1, LVEDV1, LVEF2, LVESV2 and LVEDV2

Table S2. Characteristic of different group by neutrophil and HDL-C.

Characteristic	Overall	Normal neutrophil-Normal HDL-C	Normal neutrophil-Low HDL-C	High neutrophil-Normal HDL-C	High neutrophil-Low HDL-C	P-value
N	299	110	87	75	27	
NHR	6.52 (5.09-8.44)	5.20 (4.33-6.11)	6.71 (5.47-8.48)	7.67 (6.47-8.78)	9.90 (9.27-10.51)	<0.001
Neutrophil	7.01 (5.60-8.43)	6.14 (5.25-7.04)	6.08 (4.61-6.90)	9.20 (8.59-10.14)	8.70 (8.21-9.78)	<0.001
HDL-C	1.05 (0.94-1.20)	1.13 (1.06-1.26)	0.90 (0.80-0.95)	1.23 (1.07-1.42)	0.93 (0.88-0.96)	<0.001
Time	9.00 (5.00-14.00)	9.00 (5.00-14.50)	10.00 (5.50-13.50)	8.00 (5.00-14.00)	6.00 (4.00-13.50)	0.7
LVR	81 (27.09%)	12 (10.91%)	22 (25.29%)	34 (45.33%)	13 (48.15%)	<0.001
MACE	67 (22.41%)	10 (9.09%)	18 (20.69%)	25 (33.33%)	14 (51.85%)	<0.001

High neutrophil means neutrophil ≥8×10^9/L, while Low HDL-C means HDL-C <1mmol/L.

Table S3. Associations of different group by neutrophil and HDL-C with LVR and MACE.

Exposure	Outcome	model 1	model 2	model 3
	LVR	OR (95% CI) P-value		
	Normal neutrophil-Normal HDL-C	ref	ref	ref
	Normal neutrophil-Low HDL-C	2.76(1.29, 6.17) 0.01	2.86(1.31, 6.49) 0.01	3.08(1.35, 7.31) 0.01
	High neutrophil-Normal HDL-C	6.77(3.25,14.95) <0.0001	6.87(3.26,15.38) <0.0001	7.75(3.48, 18.44) <0.0001
	High neutrophil-Low HDL-C	7.58(2.90,20.48) <0.0001	8.63(3.22,24.03) <0.0001	8.53(2.94, 25.61) <0.001
		HR (95% CI) P-value		
		model 1	model 2	model 3
	MACE			
	Normal neutrophil-Normal HDL-C	ref	ref	ref
	Normal neutrophil-Low HDL-C	2.41(1.11, 5.25) 0.0263	2.35(1.07, 5.16) 0.0343	2.34(1.04, 5.25) 0.0399
	High neutrophil-Normal HDL-C	3.58(1.72,7.47) 0.0007	3.64(1.74,7.628) 0.0006	4.54(2.07, 9.92) 0.0002
	High neutrophil-Low HDL-C	6.96(3.07,15.77) <0.0001	7.24(3.16,16.58) <0.0001	9.39(3.87, 22.76) <0.001

High neutrophil means neutrophil ≥8×109/L, while Low HDL-C means HDL-C <1mmol/L.

Model1: Unadjusted

Model2: Adjusted for Age, Smoking, Sex, and Drinking

Model3: Adjusted for Age, Smoking, Sex, Drinking, Infarction location, culprit Vessel, Hypertension, Diabetes, cTnT, Aspirin, Statin, Clopidogrel, Ticagrelor, LVEF1, LVESV1, LVEDV1, LVEF2, LVESV2 and LVEDV2

Table S4. NHR in different thrombolysis in myocardial infarction (TIMI) changes (pre-PCI to pos-PCI).

TIMI (pre-PCI to pos-PCI)	Number	NHR
0-0	1	10.37
0-2	6	8.52 (7.57-10.56)
0-3	170	6.62 (5.04-8.52)
1-2	1	6.76
1-3	42	6.09 (5.04-7.68)
2-3	34	6.28 (5.04-8.21)
3-3	45	6.35 (5.30-7.64)
Overall	299	6.52 (5.09-8.44)

NHR is expressed as median interquartile range (25%-75%).

Table S5. Net Reclassification Improvement (NRI) and Integrated Discrimination Improvement (IDI) for NHR Compared to Neutrophil Count

Metric	Value
NRI (LVR)	0.1817
IDI (LVR)	0.0216
NRI (MACE)	0.3883
IDI (MACE)	0.0424

Interpretation:

NRI:Reflects the improvement in correct reclassification of individuals (both events and non-events) when NHR is used instead of neutrophil count. Positive values indicate better reclassification by NHR.

IDI:Measures the enhancement in model discrimination ability (i.e., separation between events and non-events) achieved by incorporating NHR. Positive values indicate improved discrimination.

Table S6. Statistical Model Performance Metrics for Predicting LVR and MACE Using Neutrophil Count and NHR

Outcome	Model	AIC	BIC	Likelihood Ratio Test (*P*-value)
LVR	Neutrophil Count	482.15	492.37	
	NHR	465.92	476.13	$P < 0.01$
MACE	Neutrophil Count	392.67	402.45	
	NHR	376.20	386.42	$P < 0.01$

Interpretation:

AIC (Akaike Information Criterion): A measure of model fit, with lower values indicating better model performance.

BIC (Bayesian Information Criterion): Similar to AIC, but includes a penalty for model complexity; lower values indicate better model fit with simpler models.

Likelihood Ratio Test (P-value): Tests whether the NHR model provides a significantly better fit compared to the neutrophil count model. A significant P-value (e.g., <0.01) indicates that the NHR model outperforms the neutrophil count model.
